# Continuous sampling of cerebrospinal fluid from the third ventricle of the hypothalamus to identify accumulating or depleting metabolites during hibernation

**DOI:** 10.1242/jeb.251702

**Published:** 2026-09-09

**Authors:** Fredrik A. F. Markussen, Fernando Cázarez-Márquez, Iva Pitelkova, David G. Hazlerigg, Shona H. Wood

**Affiliations:** ^1^ Arctic Chronobiology & Physiology, Arctic & Marine Biology, BFE, UiT - Arctic University of Norway, NO-9037 Tromsø, Norway; ^2^ Proteomics and Metabolomics Core Facility, Department of Medical Biology, UiT - the Arctic University of Norway, NO-9037 Tromsø, Norway

## Abstract

Hibernation is a seasonally regulated process dramatically reducing metabolic rate and subsequently core body temperature (*T*_b_). Many hibernators repeatedly cycle between torpor and arousal (T–A cycling) throughout the hibernation season. How these cycles are regulated is still unresolved. The sensing of metabolites accumulating/depleting during torpor may regulate T–A cycling. Here, we report the development of a microdialysis protocol to sample at a high temporal resolution from the third ventricle (3V) of the hypothalamus, from an individual hibernating golden hamster (*Mesocricetus auratus*). Over multiple days and T–A cycles we saw no negative impact on hibernation physiology or dynamics during the microdialysis. We collected samples at 2 h resolution over two successive T–A cycles and measured metabolites by targeted and untargeted metabolomics. Finally, we developed an analysis pipeline to identify accumulating or depleting metabolites during torpor, providing a proof of concept that cerebrospinal fluid microdialysis from the 3V of a hibernating golden hamster can be used in future studies to understand regulation of T–A cycling.

## Teaser

**Summary:** A method to measure brain metabolites from an individual hibernating hamster over multiple torpor and arousal cycles, which could be used to investigate the regulation of hibernation.

## INTRODUCTION

Hibernation is a seasonally regulated process which results in extreme physiological changes, reducing metabolic rate up to 1% of the active state, dramatically lowering core body temperature (*T*_b_), breathing and heart rate (Lyman, 1982; Ruf and Geiser, 2015). These extreme changes in physiological state are even more remarkable because the animal repeatedly cycles between the active (aroused) and hibernating (torpid) states for the entire hibernation season (T–A cycling).

T–A cycling is a paradox: energy conservation is the ultimate purpose behind torpor, and yet hibernators undergo energy-costly periodic arousals at intervals throughout the hibernation season (van Breukelen and Martin, 2015). Much has been written on the ultimate ‘why’ reasons for this curious phenomenon (Arendt et al., 2003; Körtner and Geiser, 2000; Larkin and Heller, 1999; Pengelley and Fisher, 1961; Prendergast et al., 2002; Thomas and Geiser, 1997), but how these cycles are regulated is unresolved.

The hypothalamic circadian pacemaker in the suprachiasmatic nucleus controls daily *T*_b_ cycles in euthermic mammals and the expression of daily torpor in Siberian hamsters (Körtner and Geiser, 2000). A role for circadian control mechanisms in T–A cycling in deep hibernators is not favoured, however. Malan proposed a ‘poorly temperature compensated circadian mechanism’ in which slow running of the circadian oscillation at low temperatures times arousal (Malan, 2010; Malan et al., 2018), but this conjecture fails to account for changes in T–A cycle periodicity occurring over the hibernation season independently of ambient temperature. Furthermore, it does not account for the finding that the period of T–A cycling is the same in wild-type and tau mutant hamsters expressing a fast circadian clock (Oklejewicz et al., 2001). Current theories on the control of hibernation and T–A cycling therefore focus on the accumulation/depletion of specific metabolites/factors driving these cycles (van Breukelen and Martin, 2015). Metabolomic studies have been conducted in hibernators, and these seemingly implicate a range of metabolite signals, but there is presently no consensus on the site of action, or on the sites from which samples are taken, for example, liver (Nelson et al., 2009; Serkova et al., 2007), blood plasma (Epperson et al., 2011; Nelson et al., 2010), striatum (Osborne and Hashimoto, 2006, 2008), cistern magna (Reid et al., 1992), abdomen (Flechtner-Mors et al., 1999) and brown adipose tissue (BAT) (Fedotcheva et al., 2010). Moreover, the general use of terminal sampling techniques, and low temporal resolution of sampling hinders the interpretation required to give insight into the dynamic process of T–A cycling.

The pre-optic area of the hypothalamus and the dorsomedial hypothalamus (DMH) are key areas regulating thermoregulation in mammals (Cannon and Nedergaard, 2004; Morrison, 2016). In mice, chemogenetic and optogenetic manipulation of the neurons within the pre-optic area, some of which extend to the DMH, suppresses *T*_b_ and metabolic rate to produce a torpor-like state (Hrvatin et al., 2020; Takahashi et al., 2020). This places the hypothalamus at the centre of metabolic regulation and a candidate region for the regulation of T–A cycling.

As hibernation is a whole-animal phenomenon, it is likely that peripheral feedback to the brain is of particular importance. Peripheral feedback to the brain occurs via factors in the circulation and cerebrospinal fluid (CSF) at sites where capillaries are present or there is contact with the CSF. The median eminence located at the base of the hypothalamus is a circumventricular organ (CVO) allowing central nervous system (CNS) communication with the peripheral circulation. Tanycytes line the third ventricle (3V) of the hypothalamus, contacting the CSF, and extending processes to the median eminence and the other key hypothalamic sites such as the DMH (Langlet, 2014; Langlet et al., 2013b). Tanycytes can act as metabolic sensors and control the access of nutrients and hormones to the brain (Balland et al., 2014; Cortés-Campos et al., 2011; Dali et al., 2023; Farkas et al., 2020; Frayling et al., 2011; Langlet, 2014; Langlet et al., 2013a; Lhomme et al., 2021; Rodríguez-Cortés et al., 2022) and control seasonal changes in reproduction and metabolism by modulation of thyroid hormone signalling (Bank et al., 2015, 2017; Barrett et al., 2007; Dardente et al., 2019; Ebling and Lewis, 2018; Hanon et al., 2008; Hazlerigg and Loudon, 2008; Herwig et al., 2008; Nakao et al., 2008; Yoshimura et al., 2003). Recently, in Siberian hamsters (*Phodopus sungorus*), using a maternal photoperiodic paradigm, we identified that the numbers of sensory cilia on tanycytes alter in response to photoperiod, relating to the overwintering state of the animal (Melum et al., 2024). In the hibernating golden hamster, we also observed altered ciliary gene expression in response to seasonal status (Melum et al., 2025). Finally, we have demonstrated that tanycytes express *c-fos* mRNA, a marker of cellular activation, early in the arousal process in the golden hamster (Markussen et al., 2024). Our data suggest a seasonally regulated shift in the sensitivity of tanycytes to metabolic feedback supporting the overwintering state and that tanycytes may be responding to signals in the earliest stages of arousal from torpor (Wood, 2025). Therefore, determining what signals are available to the tanycytes in the CSF and which receptors are present on tanycytes become important questions.

To address this, it is necessary to sample CSF from the third ventricle of the hypothalamus in a hibernating hamster over a T–A cycle. However, the low brain temperature of torpor makes this technically demanding and requires the use of low flow rates to ensure the metabolites are concentrated enough to be detected. Here, we establish a protocol to sample CSF from a hibernating golden hamster at a 2 h sampling resolution and identify metabolites using reversed-phase liquid chromatography (RPLC) MS/MS. We then demonstrate a novel analysis pipeline to identify metabolites with consistent trends over two successive T–A cycles and identify which show accumulating or depleting dynamics during torpor. To explore these metabolites in relation to tanycyte receptor expression, we capitalised on a LASER capture microdissected tanycyte RNAseq dataset to identify metabolite–receptor pairs.

## MATERIALS AND METHODS

### Animals: hibernation induction and monitoring

All animal procedures were conducted at the Arctic Chronobiology and Physiology facility, UiT – The Arctic University of Norway, under license from the Norwegian Food Safety Authority (FOTS ID: 29750) in accordance with national and institutional regulations. Twenty-four, 5-month-old, male golden hamsters [*Mesocricetus auratus* (Waterhouse 1839); strain RJHan:AURA] were obtained from Janvier Labs (Le Genest-Saint-Isle, France) and housed in sibling groups for 4 weeks with food and water provided *ad libitum* at 21°C under a long photoperiod (16 h:8 h light:dark). Prior to inducing hibernation, Anipill temperature loggers (BodyCAP, Hérouville Saint-Clair, France) and IPTT-300 transponders (BMDS/Plexx BV, Netherlands) were surgically implanted under anaesthesia into the abdominal cavity and the interscapular brown adipose tissue (iBAT), respectively. Briefly, animals were deeply anesthetized with isoflurane (induction 3% and maintenance 1.8–2% isoflurane in 70% O_2_). General analgesia was injected into the lower part of the back of the animal (s.c. Meloxicam 2 mg kg^−1^ in 100 μl). After quick disinfection of the fur, the IPTT-300 (BMDS) tag was inserted subcutaneously above the iBAT using a tag injector.

For the Anipill implantation, the belly of the hamster was shaved and then sterilized with Hibiscrub and 70% ethanol. Local analgesia was injected (s.c. Lidocaine/Bupivacaine 2.5 mg kg^−1^ each in 100 μl) in the midline at the level of the incision. A small incision of the skin was made along the middle line of the abdomen (1–1.5 cm). Then, a second incision was done at the level of the linea alba. Once the Anipill was inserted, two rounds of stiches were done, a first one with resorbable thread (size: 4-0) for the muscle layer and a second one with non-resorbable thread (size: 6-0) for the skin. A 1 ml subcutaneous sterile injection of saline was given to the animals to help with rehydration as post-surgical recovery. Meloxicam was provided in the drinking bottle at a dose of 1 mg kg^−1^ for 5 days.

Following surgery, hamsters were housed individually under the same long photoperiod conditions for at least 2 weeks to ensure healthy post-operative recovery. The photoperiod was then shortened to 6 h light:18 h dark (SP-warm) at 21°C for 8 weeks to induce seasonal preparation for hibernation.

### Craniotomy, guide cannula implantation

After 8 weeks in SP-warm conditions, hamsters (body mass 132.3±5.7 g; mean±s.d.) were deeply anesthetized in isoflurane (3% induction, 1.8–2% maintenance in 70% O_2_). General analgesia was injected in the lower part of the back of the animal (Meloxicam 2 mg kg^−1^ in 100 μl). Local analgesia was injected under the scalp (Lidocaine/Bupivacaine 2.5 mg kg^−1^ in 100 μl). Hamsters were placed on the stereotaxic apparatus (Kopf Model 902) coordinates for guide cannula insertion were calculated according to the golden hamster brain atlas (Morin and Wood, 2001). A hole of 2 mm was drilled at −0.7 mm anterio-posterior and 0.0 mm medio-lateral. The guide cannula (CMA11) was first introduced 0.5 mm lateral to the venular superior sagittal sinus to cross the dura mater, gently pushing the superior sagittal sinus to the side while positioning the cannula in the 0.0 mm medio-lateral position and inserting to a depth of 8 mm (later modified to 7 mm). Guide cannula and a head screw (CMA) were secured to the skull using two anchor screws and dental cement (Superbond). Post surgery, an injection of buprenorphine (0.03 mg kg^−1^ in 100 μl) was administered as well as 1 ml of sterile injectable saline once the animal was about to be removed from the isoflurane anaesthesia. Meloxicam was given in the drinking bottle in a dose of 1 mg kg^−1^ for 5 days. Hamsters were allowed to recover for at least of 10 days post-surgery before brain microdialysis. Finally, the ambient temperature was reduced to 6°C (SP-cold) to induce hibernation and T–A cycling.

### Microdialysis and fraction collection from hibernators

We waited until hamsters displayed at least two T–A cycles and were euthermic before threading the animals with a microdialysis probe. Microdialysis probes with two molecular size cut-offs were used: CMA 11, 6 kDa, 2 mm (8309482) or CMA 11, 55 kDa, 2 mm (8012512). To allow free movement of the animal, a swivel was attached to a multi axis counterbalance lever arm (Instech, MCLA 61-0023). Microdialysis was performed with sterile artificial cerebrospinal fluid (aCSF, CMA, 59-7316) delivered by a 10 ml PPE syringe (B Braun, 4606108 V) using a programmable pump (Aladdin SyringeONE, AL-1000). Following the CMA Harvard apparatus User's Manual, the microdialysis probes were mounted and fixed to the CMA 11&12 Probe clip on the CMA/130 *in vivo* stand (CMA8309102), placing the probe tip in a vial filled with aCSF. Then with the use of adapters (CMA, 3409500), the probe's inlet and outlet with the swivel (Instech, 375/D/22QE), were connected using 2×40 cm FEP tubing (CMA, 8409501) flushing with aCSF at a rate of 10 μl min^−1^ for 5 min while tapping the probe clip with a tweezer to remove bubbles. After verifying the absence of bubbles, the pump was set to 1 μl min^−1^.

Hamsters were anesthetized under isoflurane (3% induction, 1.8–2% maintenance in 70% O_2_) and maintained warm with the heat pad. The dummy cannula was removed from the guide cannula and then without stopping the flow of the aCSF, the prepared microdialysis probe was inserted and the system was anchored to the hamster's head with screws, leukoplast® and glue. The whole system and animals were placed in their home cage with a modified lid (Eurostandard, Type III) (Fig. S1A).

Once the animals were tethered, the microdialysis probe was left running for 3 days (72 h) at 1 μl min^−1^ to equilibrate. On the fourth day, collections were made every 2 h at a volume of 120 μl. Fractions were collected using the automatized CMA 470 Refrigerated fraction collector to keep the samples at 6°C. Samples were removed from the fraction collector approximately every 12 h and frozen at −20°C (1 month) then transferred to −80°C for long term storage. Hamsters were microdialysed for up to 12 days and then euthanised with an overdose of isoflurane (5% in air) and cervically dislocated.

### Confirmation of probe placement

**Fig. 1.**
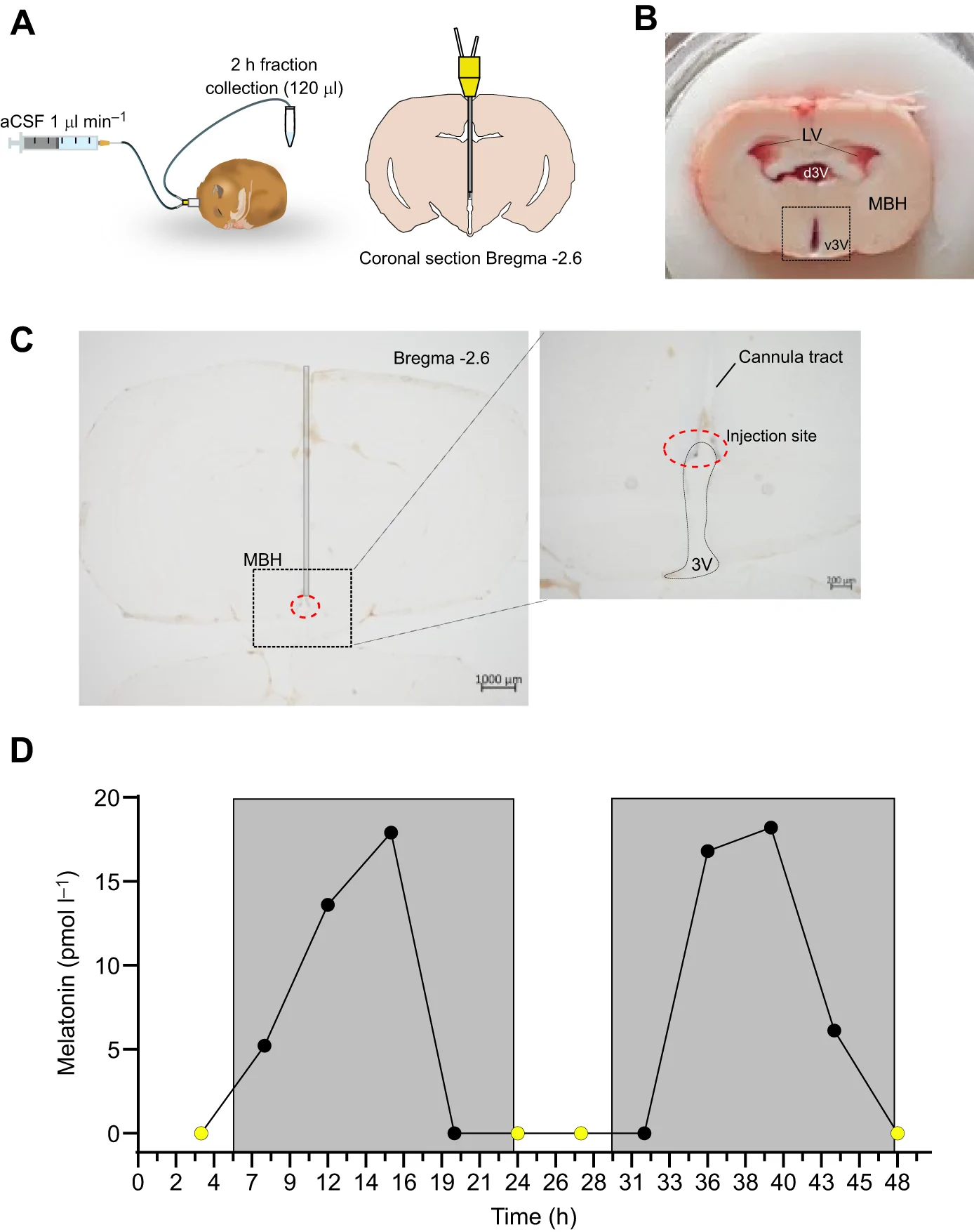
**Successful CSF sampling from the third ventricle of the hypothalamus of the golden hamster (*Mesocricetus auratus*).** (A) Schematic of the microdialysis sampling procedure along with the coordinates for the probe placement. Microdialysis was performed with sterile artificial cerebrospinal fluid (aCSF). (B) Coronal section of a hamster brain cut on a cryostat showing cresyl violet staining in the third ventricle confirming probe penetration. (C) Nissl-stained coronal sections of a hamster brain showing the area penetrated by the cannula. (D) Melatonin concentration in pmol l^−1^ from CSF sampled during the dark (black dots and grey boxes) and light phase (yellow dots) from one animal over 2 days. LV, lateral ventricles; MBH, mediobasal hypothalamus; v3V, ventral third ventricle; d3V, dorsal third ventricle.

Brains were dissected taking care that the microdialysis probe and the guide cannula did not damage the placement area in the brain. Coronal brain sections (20 μm) were cut using a cryostat (CM3050 S, Leica) and collected directly on Superfrost plus slides. One in every 6 coronal sections was collected through the whole mediobasal hypothalamus (MBH) (from 0.5 to –3.6 mm using Bregma as a reference in the hamster brain atlas (Morin and Wood, 2001). Nissl staining with Cresyl Violet (0.5% w/v) was performed to counterstain and localise the probe tip. Fig. 1A–C shows the placement of the cannula for hibernating animals. Fig. S1B shows the cannula placement for all animals and Fig. 1B,C shows the successful placement of the cannula.

### CSF melatonin measurement

Melatonin was measured using mass spectrometry as previously described (van Faassen et al., 2017). Briefly, 25 μl CSF microdialysate, 25 μl melatonin-D4 internal standard solution and 100 μl 0.04% ascorbic acid were added to wells of a 700 μl polypropylene 96-deep well plate (Waters). The plate was sealed before being vortex mixed for 10 min. Following vortexing, the plate was transferred to the autosampler and 50 μl was injected for analysis. Online solid phase extraction (SPE) was performed using the fully automated Chronect Symbiosis Advanced system (Axel Semrau, Sprockhövel, Germany). Oasis HLB (Waters, Milford, USA) cartridges were used for extraction. Liquid chromatography (LC) was performed on a Phenomenex Luna Phenyl-Hexyl 2.0×100 mm, 3 μm column (Phenomenex, Torrance, CA, USA) and total run time was 6.5 min, including online SPE cleanup. Melatonin was analysed in positive ionization mode on a Xevo™ TQ Absolute triple quadrupole mass spectrometer (Waters, Milford, USA).

### RPLC untargeted metabolomics

One hundred samples were selected from a single representative hamster across two torpor bouts, from which the probe placement was in the third ventricle. We used reversed-phase liquid chromatography (RPLC) with positive electrospray ionization (RPLC ESI^+^) (Grijseels et al., 2024). Briefly, each sample was extracted in an ice-cold acetonitrile:methanol mixture (−20°C) under agitation, then injected into a Thermo Fisher Scientific Vanquish Horizon UHPLC system coupled to an Orbitrap ID-X Tribrid mass spectrometer. Chromatographic separation was done on an HSS T3 column (150×2.1 mm; 0.18 nm) with a 19.6 min gradient of aqueous ammonium formate (mobile phase A) and acetonitrile (mobile phase B) at 0.35 ml min^−1^. One microlitre of each sample was collected for pooled quality (QC) controls and were injected periodically (approximately every 10 samples). Pooled sample metabolite abundances were used to normalize sample abundance values and to assess instrumental drift.

Raw data were processed using Compound Discoverer 3 (Thermo Fisher Scientific) and features were identified based on mass, retention time and MS/MS spectral similarity against mzVault (UiT - Proteomics and Metabolomics Core Facility in-house database; Myhre et al., 2026), mzCloud (ThermoFisher) and ChemSpider (open data) databases. Metabolite annotation followed a modified Metabolomics Standards Initiative (MSI) confidence level annotation (Schymanski et al., 2014; Sumner et al., 2007), where level A requires MS/MS spectral and retention-time matches with in-house standards, level B requires ≥60% spectral match in mzCloud, levels C–D indicate putative annotations (C: match in mzCloud 0–60% or D: ChemSpider-only matches), level E covers metabolites with a MZCloud similarity match and level F to indicate unknown features (predicted composition only).

### Untargeted statistical analysis

Data acquired from the RPLC ESI^−^ MS/MS was analysed using R v.4.3.3. We included all metabolites with a predicted composition (MSI A-F) and a measured mass within 5 ppm of the predicted composition mass. Scripts and data used for analysis are available in our GitHub repository (https://github.com/fredrik-markussen/Torpor-CSF-UntargetedMS). Prior to statistical analysis, metabolite abundance was normalized against pooled QC samples to correct batch effects and instrument drift. We then square-root transformed the abundances to effectively handle zero and near zero values while compressing the effect of large values, followed by centring around zero and scaling by subtracting the mean and dividing by the standard deviation to standardise across metabolites (z-normalization).

In brief, to identify accumulating and depleting metabolites during torpor we had a three-step filtering approach. (1) Identify metabolites cross correlating with *T*_b_ and select those with a significant (*P*<0.05) Spearman's rank correlation coefficient (ρ). (2) Assess the consistency of the relative abundance changes of *T*_b_ correlating metabolites between T–A cycles, selecting metabolites with an interclass correlation coefficient (ICC) greater than 0.4._._ (3) Perform a Hamed–Rao modified Mann–Kendall test on the metabolites from (2) to assess whether there are any significant accumulating or depleting trends during torpor (Benjamini–Hochberg FDR<0.05).

#### Identify metabolites cross correlating with *T*_b_

Cross-correlation takes two time series and systematically shifts one relative to the other to evaluate their correlation at various lag positions. This approach identifies the time delay (lag) at which the correlation between the series is maximized, revealing potential lead-lag or synchronous relationships between metabolite and *T*_b_. The relationship between each metabolite abundance and *T*_b_ was tested using Spearman's rank correlation coefficient (ρ) over a lag interval covering half the period of one of the two investigated T–A cycles (104/2=52 h). To account for the high dimensional and cyclical nature of the data in which correlation may not be linear, we applied a permutation-based Spearman's correlation test to evaluate the statistical significance of correlation at each lagged position (Liu et al., 2018; Yu and Hutson, 2024). Specifically, the temporal order of *T*_b_ was randomly shuffled (permuted) 1000 times, generating the null distribution of correlation values at each lag position. From this distribution of Spearman's rho, the 99% confidence interval (CI) could be calculated at each lag position. We then filtered significant but weak or potentially spurious correlations by only retaining a metabolite when there were at least five sequential lag positions that were over the 99% CI. This conservative approach ensured robustness of the correlation and is based on the premise that a significant metabolite will show an increasing strength of correlation prior to its ‘peak’ correlation at a specific lag (example of the output is shown in Fig. S2A,B). Thus, the cross-correlation approach reflects the aggregate relationship between metabolite abundance and *T*_b_ across the two T–A cycles. Therefore, correlations only found in one T–A cycle should fail the permutation significance testing, increasing the likelihood that the reported significant metabolites are consistent across both T–A cycles.

To visualise data, we used the period length of the second T–A cycle (104 h) to estimate phase for each metabolite. We converted phase into 360 deg to represent the cycle; therefore, for each metabolite, the lag hour for the peak of cross-correlation was divided by the period length multiplied by 360. This approach aids visualisation and allows interpretation of the dynamics of metabolites relative to the *T*_b_ changes observed. For example, if the metabolite directly follows *T*_b_, the phase of the metabolite will be displayed at 0 deg.

#### Interclass correlation coefficient (ICC)

To directly compare metabolite dynamics between two T–A cycles and statistically test for trends, we first converted bout hours to percentages from 0 to 100% torpor bout completion. A 0% torpor bout completion was defined as the animal passing 30°C in the entry phase and 100% torpor bout completion defined as the beginning of rewarming, i.e. an acute *T*_b_ increase of >1°C. Percentage numbers for *T*_b_ and CSF sample points are then assigned by generating a sequence from 0 to 100 aligned to the number of samples available within the window defined above. To make bout 1 and bout 2 directly comparable to each other, *T*_b_ and sample points were linearly interpolated on to a standardised 0–100% grid using a 2.5% step size. To facilitate calculation of the ICC score, we removed outliers using a >3 s.d. threshold in a simple linear regression model, replacing the outlier by the mean of the two flanking nearest neighbours, maintaining sample numbers. The ICC score between bout 1 and bout 2 was then calculated using a two-way mixed effects single measurement model rating consistency (Koo and Li, 2016). After evaluating the distribution of ICC scores and the metabolite profiles from both T–A cycles (Fig. S2C), we classified the consistency of the metabolites between the bouts as follows; ICC≤0.4 as poor, ICC 0.4–0.65 as moderate, ICC 0.65–0.9 as good and ICC≥0.9 as excellent based on Koo and Li (2016) (Fig. S2C).

#### Modified Mann–Kendall test

The Hamed–Rao modified Mann–Kendall trend analysis tests for time-series trends while correcting for autocorrelation (Hamed and Ramachandra Rao, 1998). The modified Mann–Kendall is especially suited to test for accumulating and depleting metabolites because it tests for monotonic trends, does not assume linearity, handles non-normally distributed data and can correct for autocorrelation in timeseries data, addressing the major problems with the cross-correlation analysis we performed earlier. The modified Mann–Kendall computes tau (τ), indicating strength and direction of the trend, and Sen's slope indicating average rate of change across the torpor bout. We computed τ for CCF significant metabolites using the median value from bouts 1 and 2 (Khambhammettu, 2005) and corrected *P*-values for multiple testing using Benjamini–Hochberg correction at a false discovery rate (FDR) of 5%. A descriptive monotonic trend shape classification was made, dividing monotonic trends into ‘elbow-early’ defined as 50% total range change before 20% torpor bout completion, ‘elbow-late’, defined as 50% total range change of 20–30% torpor bout completion and ‘near-linear’ as 50% total range change of 30–70% torpor bout completion. ‘Reverse-elbow’ patterns were also classified, defined as 50% total range change after 70% torpor bout completion but no significant patterns were found. All data and R analysis pipeline are available through our GitHub repository (https://github.com/fredrik-markussen/Torpor-CSF-UntargetedMS).

### Tanycyte receptor–metabolite pairing

RNA-seq data generated from golden hamster LASER captured tanycytes (Melum et al., 2025) (GEO accession identifier: GSE281814) provided gene expression data for this analysis. In our previous study we sampled animals in long and short photoperiods at different stages of the hibernation season (Melum et al., 2025); here, we determined the median counts per million (CPM) across the whole experiment for each gene and applied a cut-off of 10 raw counts, removing genes with a median of less than 0.5 CPM from the analysis. 16,315 genes remained representing our tanycyte enriched transcriptome. We cross-referenced this with the MRCLinkdb (Zhang et al., 2024) metabolite receptor database for both humans and mice. MRCLinkdb has established known metabolite, receptor, transporter and enzyme pairings for 316 metabolites. We plotted the matching golden hamster metabolites found in the MRCLinkdb with the listed receptor, transporter and enzyme genes and the CPM for each gene from the golden hamster tanycyte-enriched transcriptome and the metabolites showing cross correlation with *T*_b_, an ICC greater than 0.4 and an FDR less than 0.05 in the modified Mann-Kendal test.

## RESULTS AND DISCUSSION

### Sampling of CSF from the third ventricle of non-hibernators

As a first proof of principle, freely moving golden hamsters housed in short photoperiod (SP) at 21°C were attached to the microdialysis tether system at a flow rate of 1 μl min^−1^ (Fig. 1A) and sampled continuously. Post-mortem we verified our bregma coordinates were correct by injecting Cresyl Violet stain and cryosectioning the brain to show that the third ventricle was penetrated (Fig. 1B). Based on the results from the first four animals we adjusted the depth to 7 mm from 8 mm to avoid damage to the median eminence ventricular floor and dialysate probe tips. This improved our success at probe placement in the subsequent seven animals (Fig. 1C). The modified coordinates prevented damage to the 55 kDa probes, unfortunately, the 6 kDa probes were more delicate and still tended to be damaged, potentially during insertion. Prior to death we used the microdialysis system continuously sampling for up to 12 days. No changes in behaviour, food intake or weight were observed.

We collected CSF fractions every 2 h (maximum volume per fraction 120 μl). To ensure that the samples were concentrated enough while using this flow rate and sampling frequency, we performed mass spectrometry measurements for melatonin on 12 fractions sampled 4 h apart covering 48 h. This showed that melatonin peaks in concentration in the dark phase at 18 pmol l^−1^ and is below the detection limits (<5 pmol l^−1^) in the light phase (Fig. 1D), confirming our sampling scheme was valid.

### Hamsters initiate hibernation and continue to T–A cycle throughout CSF microdialysis

Twelve animals were moved into SP at 6°C to initiate hibernation, seven had guide cannulas implanted. Only one cannula-implanted animal failed to initiate hibernation, therefore our hibernation rate was 91.6%, which is similar to previously reported results (Markussen et al., 2024).

**Fig. 2.**
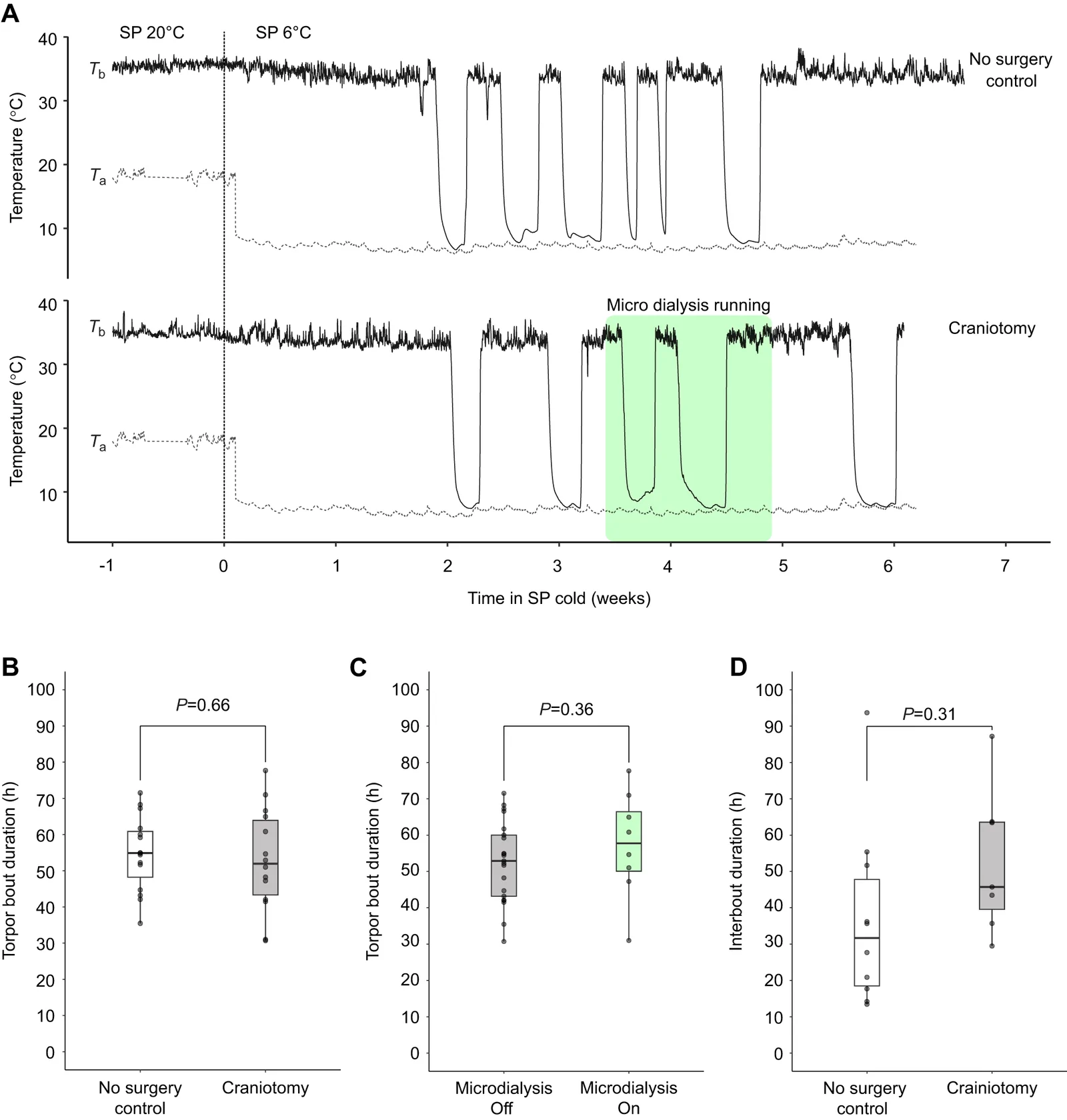
**Microdialysis has no impact on golden hamster hibernation.** (A) Body temperature profiles (*T*_b_) from an animal that did not have brain surgery (no surgery control; top graph) and one that had a craniotomy and was microdialysed (craniotomy; bottom graph). The green shading represents when microdialysis occurred. SP, short photoperiod; *T*_a_, room temperature. (B) Comparison of torpor bout duration for no surgery animals (white box) and craniotomy animals (grey box). Each dot represents torpor bout length in hours from 2–3 bouts per animal with crainiotomy. *t*-test showed *P*=0.66. (C) Comparison of torpor bout duration for craniotomy animals when the microdialysis was on (green box) or off (grey box). Each dot represents torpor bout length in hours from 1–3 bouts per animal. *t*-test showed *P*=0.36. (D) Comparison of interbout duration for no surgery animals (white box) and craniotomy animals (grey box). Each dot represents interbout length in hours from 1–2 bouts per animal (*P*=0.31, *t*-test). Boxes show the 25–75th percentiles with median; whiskers show the 1.5× interquartile range. *N*=6 (crainiotomy), *N*=5 (no surgery).

We saw no impact of craniotomy or microdialysis on the body temperature profiles of the hibernators (Fig. 2A). We also saw no difference in the torpor bout duration or inter bout euthermy duration between craniotomy, microdialysed and untreated animals (Fig. 2B–D). We successfully microdialysed from all six hibernating animals for up to 2 weeks. However, post mortem, we found that only three of the probes were completely in the third ventricle.

### Hibernating hamster CSF metabolome shows clear cycles of metabolites throughout the T–A cycle

**Fig. 3.**
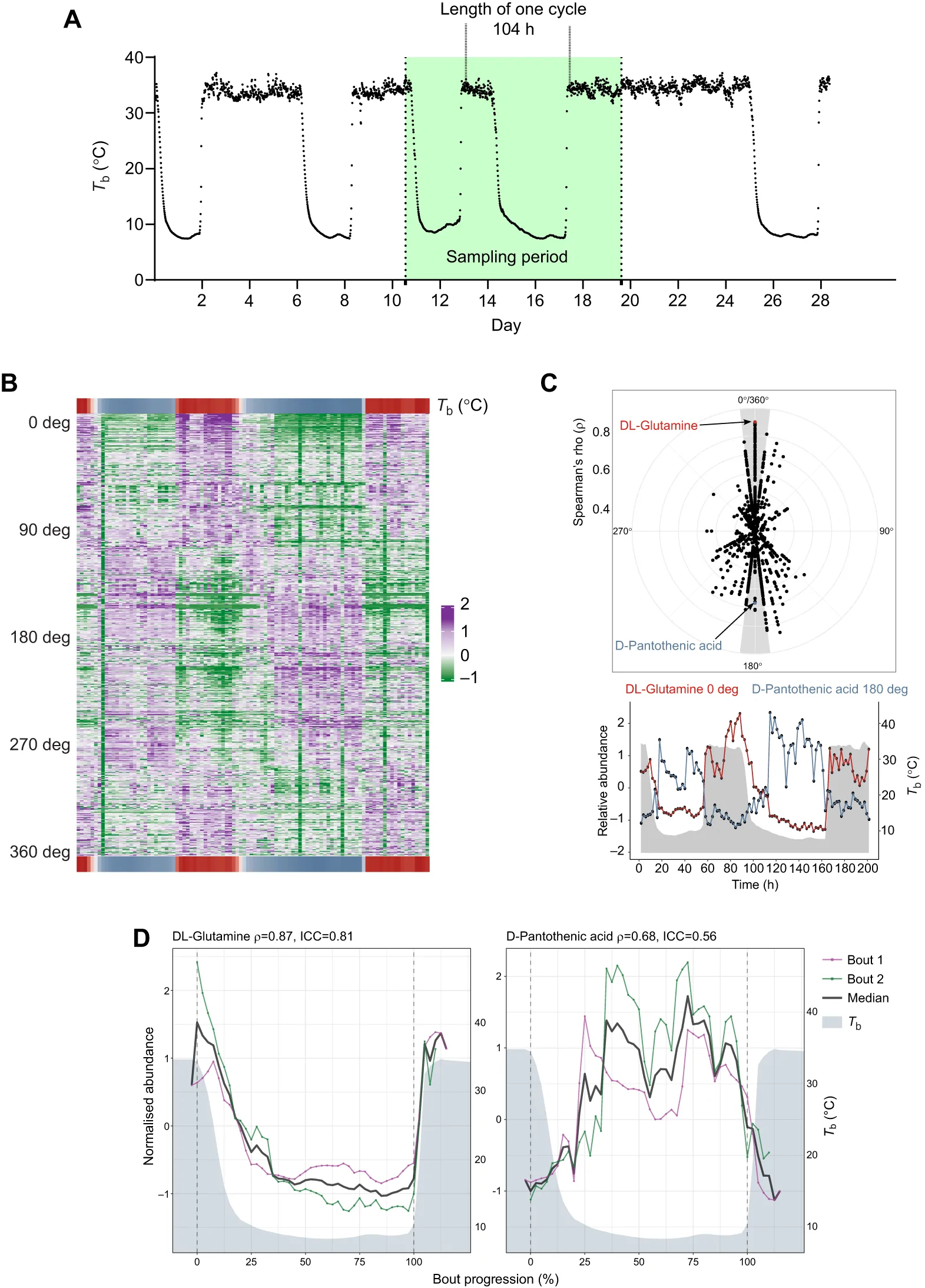
**Relative abundance of CSF metabolites from a hibernator correlates with body temperature.** (A) Body temperature (*T*_b_) profile from one animal selected for metabolomic analysis. The sampling period is indicated in green. (B) Heatmap of the 761 metabolites that are significantly cross-correlated with body temperature. Body temperature (*T*_b_) is displayed at the top and bottom of the heatmap, red for warm and blue for cold. The relative abundance of each metabolite is displayed on a colour scale from −1 (green) to 2 (purple). The metabolites are ordered according to the phasing of the cross correlation in relation to body temperature, calculated on a 360 deg basis to represent a cycle (based on the period length of the second T–A cycle, shown in A). (C) Top: circular plot showing each cross-correlated metabolite's Spearman's rank correlation coefficient (ρ) (the further from the centre of the plot, the more strongly correlated). The position of the metabolite on the circle represents the phase of metabolite in relation to body temperature, calculated on a 360 deg basis to represent a cycle (based on the period length of the second T–A cycle, shown in A). Grey shading shows the ±2 h sampling window around metabolites in phase with *T*_b_ (0/360 deg) and in antiphase (180 deg). Bottom: an example of 0 and 180 deg metabolites in a relative abundance plot of DL-glutamine in red and D-pantothenic acid in blue over the 200 h sampling period. The animal's body temperature (*T*_b_) is displayed as the grey shaded area marked the right *y*-axis. (D) Relative abundance traces across the two torpor bouts converted to percent completed torpor. The two metabolites are in opposite phase. DL-glutamine (left, Spearman's correlation coefficient, ρ=0.87, interclass correlation coefficient, ICC=0.81) and D-pantothenic acid (right, ρ=0.68, ICC=0.56). Black trace shows the median value of the bout 1 (pink line) and bout 2 (green line) and the animal's median body temperature (*T*_b_) is displayed as the grey shaded area, *n*=1.

To assess the metabolomic changes during a T–A cycle, we selected one animal where we had successfully sampled continuously for two full T–A cycles and performed RPLC on these 100 samples, corresponding to 200 h of microdialysis (Fig. 3A).

We detected 1312 metabolites (MSI level A–F), in the CSF determining the relative abundance across two T–A cycles. We then performed a cross-correlation analysis to determine the relationship between relative abundance and recorded *T*_b_. To conduct this analysis, we used the cycle length of our second T–A cycle, starting 1 h after IBE from the previous torpor bout and finishing 1 h after IBE from the second torpor bout, this was 104 h (Fig. 3A); therefore the maximum lag time in our analysis was 52 h. A metabolite was determined to be significantly correlated to *T*_b_ based on the 99% confidence interval from the cross-correlation analysis. Over half of the metabolites (761) showed a significant cross-correlation with body temperature (Table S1). To visualize the phasing of the metabolites as a cycle, we used the cycle length of the second T–A cycle and converted time into 360 deg. This means that metabolites with a significant cross correlation and a phase of 180 deg are increased in abundance during torpor (when *T*_b_ is low) in both T–A cycles. Whereas metabolites with a significant cross correlation and a phase of 0 deg are decreased in abundance during torpor in both T–A cycles (Fig. 3B,C). The 761 cross-correlated metabolites are presented in a heatmap ordered by phase and maximum correlation (Fig. 3B). Using a ±2 h window based on the sampling frequency we determined that 55% of the significantly correlated metabolites were either ‘in phase’ with body temperature (0 deg, 238 metabolites, DL-glutamine shown in Fig. 3C) or ‘in antiphase’ to body temperature (180 deg, 184 metabolites, D-pantothenic acid shown in Fig. 3C).

**Fig. 4.**
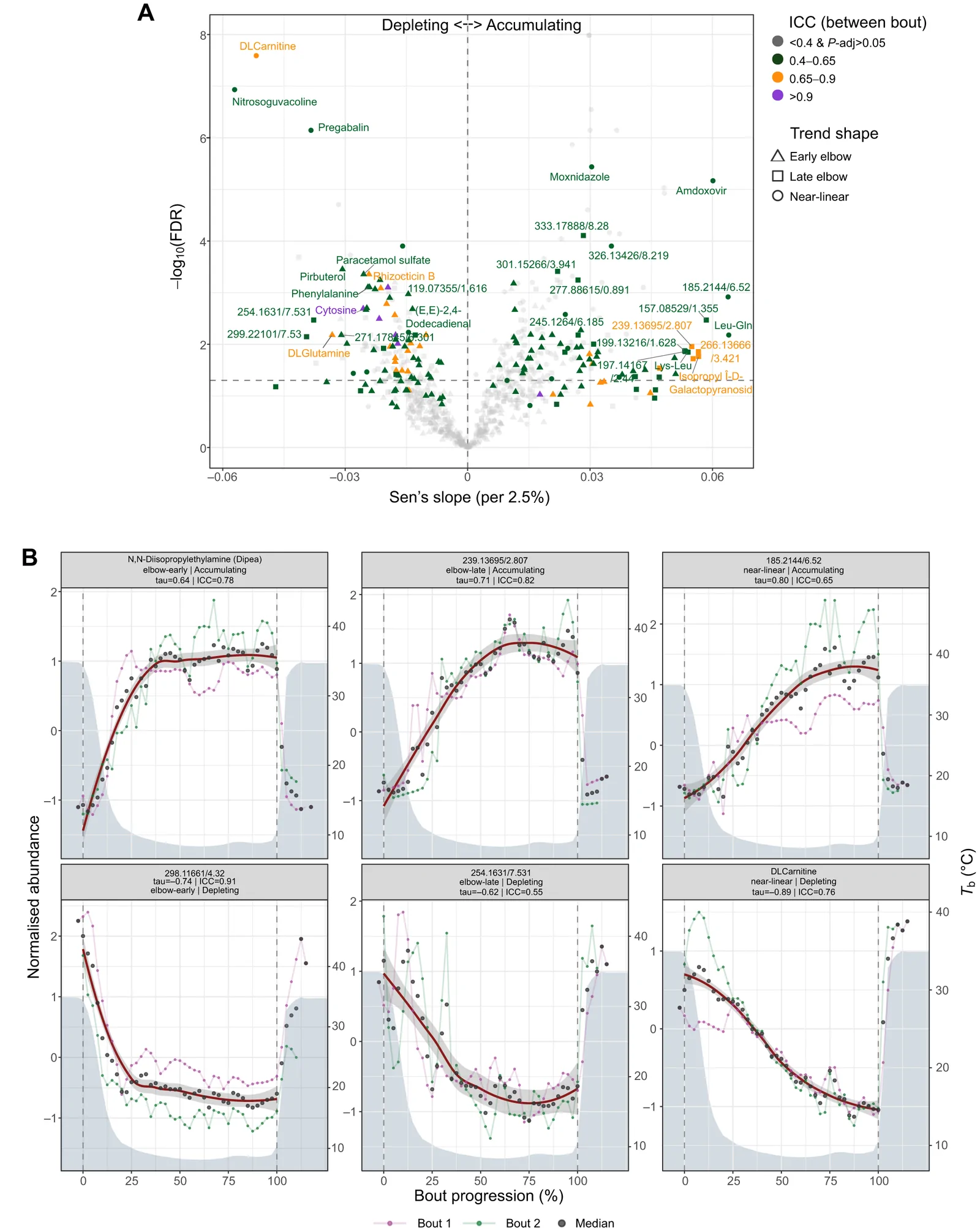
**CSF metabolites deplete and accumulate across torpor in the third ventricle.** (A) Volcano plot showing the distribution of the Mann–Kendall trend results. The Benjamini–Hochberg corrected false discovery rate (FDR) from the Mann–Kendall trend test is displayed as –log_10_ on the *y*-axis and Sen's slope indicating the average rate of change across the torpor bout is displayed on the *x*-axis. In the simplest interpretation, negative Sen slope indicates a depletion and a positive value indicates an accumulation during torpor. Each metabolite is coloured according to the interclass correlation coefficient for the consistency between bouts. The symbols indicate the trend as early or late elbow or near linear. The top 30 metabolites [lowest FDR *P*-value and highest Mann–Kendal tau (τ)] are labelled by name. (B) The metabolites with the highest τ and the highest interclass correlation coefficient (ICC) for each of our defined trends. Elbow-early accumulating: *N*,*N*-diisopropylethylamine [Dipea, τ=0.64, ICC=0.78, Benjamini–Hochberg FDR (*P*-adj)=0.004]; elbow-early depleting: 298.11661/4.32 (τ=−0.74, ICC=0.91, *P*-adj=0.001); elbow-late accumulating: 239.13695/2.807 (τ=0.71, ICC=0.82, *P*-adj=0.017); elbow-late depleting: 254.1631/7.531 (τ=−0.62, ICC=0.55, *P*-adj=0.005); near-linear accumulating: 185.2144/6.52 (τ=0.80, ICC=0.65, *P*-adj=0.002); near-linear depleting D/L-carnitine: (τ=−0.89, ICC=0.76, *P*-adj≤0.001). Solid red line indicates fitted model (loess) with shaded standard error area used for shape determination. Bout 1 is the pink line and bout 2 is the green line. The animal's median body temperature (*T*_b_) is displayed as the grey shaded area, *n*=1.

Whilst this approach can identify metabolites that are cross-correlated with *T*_b_ its estimation of between T–A bout consistency is not direct and metabolite relative abundance trends showing accumulation and depletion during torpor are not easily identified. Therefore, we took the 761 cross-correlated metabolites and directly compared the two T–A cycles by converting time to percentage completed torpor and then calculated the median value for each cross-correlated metabolite across both T–A cycles (Fig. 3D). We used the interclass correlation coefficient (ICC) to filter the metabolites based on their consistency between bouts; of these, 195 metabolites had an ICC above 0.4, indicating that they behaved consistently across both torpor bouts (Table S1, Fig. S2C). We then performed a trend analysis (modified Mann–Kendell) to identify which of the 195 metabolites showed a depleting or accumulating trends during torpor and found that 127 out of the 195 bout consistent metabolites showed a significant trend during torpor (Benjamini Hochberg FDR <0.05; Table S1, Fig. 4A). We subsequently categorized these as either a ‘near-linear’ trend (7 depleting, 9 accumulating), an ‘elbow-early’ trend (49 depleting and 38 accumulating) or an ‘elbow-late’ trend (6 depleting, 18 accumulating) (Fig. 4A,B). The defining feature of the elbow-early trend is a rapid change in metabolite relative abundance during the period of most rapid cooling (entry to 20% torpor), suggesting that these metabolites may be closely following body temperature and once the torpor temperature is relatively stable, the abundance of the metabolite also stabilizes, until rewarming (Fig. 4B). The elbow-late trend was defined as the most rapid changes in abundance occurring after 20% and before 30% completed torpor, therefore they did not directly follow the *T*_b_ decline but did appear to stabilise after 50% completed torpor (Fig. 4B). Finally, the near-linear trend (defined showing the largest changes in metabolite abundance within 30–70% torpor completion window) showed a clear accumulation or depletion across torpor which ‘recovered’ during interbout arousal, DL-carnitine is the most significant example (Fig. 4B). It is important to note that the metabolomics assay used cannot establish chirality, as D-carnitine is not endogenously produced, it is probable that this is L-carnitine; therefore, we refer to it as L-carnitine from here on.

**Fig. 5.**
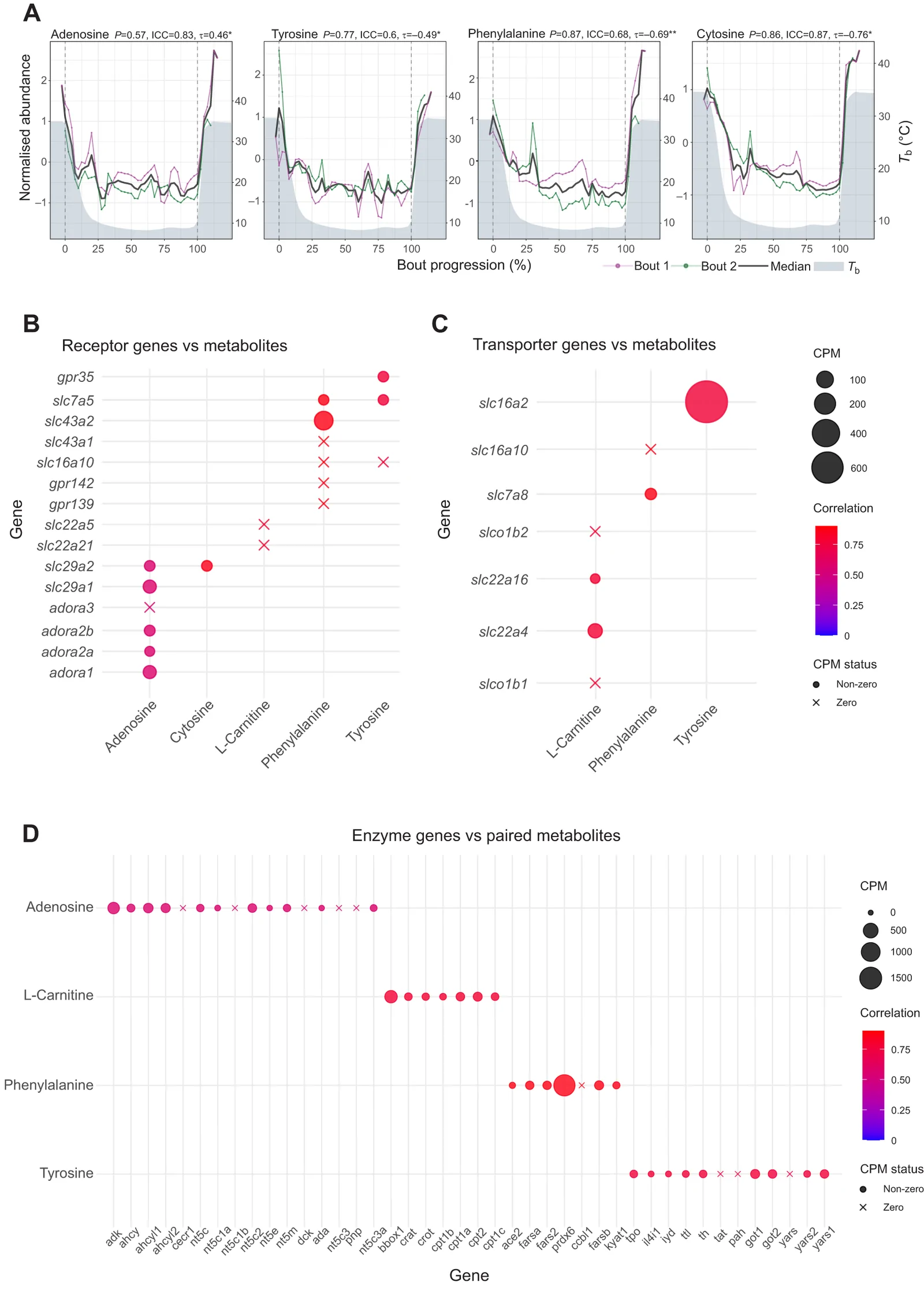
**Tanycytes express receptors and transporters for CSF metabolites that accumulate and deplete during hibernation.** (A) Relative abundance plots for adenosine (MSI A), tyrosine (MSI A), phenylalanine (MSI A) and cytosine (MSI D) across the two torpor bouts standardised to percentage completed torpor. Black trace shows the median value of the bout 1 (pink line) and bout 2 (green line) and the animal's median body temperature (*T*_b_) is displayed as the grey shaded area, *n*=1. (B) Dot plot showing the metabolites in relation to their reported receptors. The dot size represents the counts per million of each gene from a tanycyte enriched RNA-seq analysis. The colour shows the strength of the cross-correlation of the metabolite with body temperature. X indicates that the gene is not expressed in the tanycyte enriched RNA-seq dataset. (C) Dot plot showing the metabolites in relation to their reported transporters. (D) Dot plot showing the metabolites in relation to their enzyme genes. CPM, counts per million.

The identity of many of our significant metabolites is presently difficult to establish definitively. The annotation of metabolites relies on matching mass spectra to known standards. If there is a good match to our local in-house metabolite spectral library, the metabolite is defined as level A, the weaker the match the lower the level (see Materials and Methods, MSI A–F). Therefore, only those metabolites identified as level A can be definitively identified. Unfortunately, many metabolites cannot be definitively identified (5/1312 metabolites were MSI level A). This highlights the need for better annotation and cautious interpretation results from putative identifications. Amongst our 127 accumulating/depleting metabolites (ICC greater than 0.4, Mann–Kendall BH *P*-value less than 0.05) there were only three MSI level A matches; adenosine, phenylalanine and tyrosine (Fig. 5A).

### Tanycytes lining the third ventricle have receptors for cycling CSF metabolites

We were interested to know whether any of our T–A cycle changing metabolites were ligands for tanycyte receptors, or whether tanycytes contained enzymes or transporters for these metabolites. We used a metabolite receptor interactor database (MRCLinkdb) (Zhang et al., 2024) for both humans and mice that has established known metabolite receptor pairings for 316 metabolites. To cross-compare to this database, we used all the identified metabolites (1312) with a HMDB identifier regardless of correlation (229) and found 16 were present in the MRCLinkdb; 13 of these 16 were metabolites that cross-correlated with body temperature and of these, five showed a significant trend and an ICC >0.4: adenosine (MSI level A), L-carnitine (MSI level C: DL-carnitine; Fig. 4B), tyrosine (MSI level A), phenylalanine (MSI level A), cytosine (MSI level D) (Fig. 5A).

Using the MRCLinkdb we identified the known receptors, enzymes and transporters of the five significant golden hamster CSF metabolites. Then, we used our existing RNAseq dataset from LASER captured golden hamster tanycytes (Melum et al., 2024) to see whether any of the receptors, enzymes or transporters were expressed. We found that golden hamster tanycytes express one or more receptors or transporters for our five metabolites (Fig. 5B,C). We noted that out of the six known adenosine receptors, five were expressed in our tanycyte enriched transcriptome, along with 11 out of 16 enzymes involved in reactions with adenosine (Fig. 5D).

Enzymes involved in the reactions of four of the significant metabolites were expressed in tanycytes (Fig. 5D), including L-carnitine which shows a significant depletion during torpor (Fig. 4B). Tanycytes also appear to express 2 out of 4 transporters for L-carnitine (Fig. 5C). Tyrosine and phenylalanine (Fig. 5A), also have tanycyte expressed receptors, transporters and enzymes (Fig. 5A,C,D).

### Concluding remarks

Here, we have shown that it is possible to sample CSF from the third ventricle of a freely moving and hibernating golden hamster. We show that the welfare of the animals is maintained, and the dynamics of T–A cycling are not impacted by microdialysis. However, we note that the success at correctly positioning the probe in the third ventricle was only 44% (Fig. S1B), highlighting the importance of postmortem validation of probe placement for the interpretation of the results. The current golden hamster brain atlas was validated with stereotaxic surgeries and notes that the coordinates are within 0.5 mm of the target sites (Morin and Wood, 2001), given the fact that the third ventricle is only 0.2 mm wide, it is perhaps not surprising that our success rate was only 44%. While sampling from the lateral ventricle would have been easier and likely resulted in a higher success rate our hypothesis was that the tanycytes may be the major communication relay to the thermoregulatory circuits of the hypothalamus from the CSF, therefore we had to focus on the third ventricle.

Previously, it has been noted that microdialysis in hibernators should be conducted at a low flow rate to prevent effects of temperature on the diffusion across the membrane (Osborne and Hashimoto, 2006) (Prof. Kelly Drew, The University of Alaska, Fairbanks, pers. comm.). Our flow rate of 1 μl min^−1^ resulted in the preliminary detection of a total of 1312 metabolites, which is in line with similar human studies of CSF (Sindelar et al., 2018). Also, the observation that not only did metabolites decrease in relative abundance during torpor but also increased, argues against any temperature effects on the permeability and diffusion across the microdialysis membrane. Therefore, we assert that the 761 metabolites cross-correlated to body temperature, some with different phase relationships to the body temperature, are biologically relevant changes.

We have shown that our analysis pipeline, can be used to identify consistent significant accumulating or depleting metabolites during torpor across two T–A cycles. In this proof of principle study, the two T–A cycles measured are from the same animal, this limits the interpretation of the identified accumulating and depleting metabolites. However, the analysis pipeline we present can be used in the future to assess inter-animal as well as inter-bout consistency and select appropriate candidate metabolites for follow studies.

Our previous work showed that tanycytes may have a role to play in T–A cycling (Markussen et al., 2024) and that the number of sensory cilia on tanycytes alters according to seasonal status (Melum et al., 2024). If a change in metabolite status in the CSF of the third ventricle was an important signal in regulating the T–A cycle it could be sensed by the tanycyte. To explore this we matched receptors, enzymes and transporter genes from golden hamster tanycytes to our CSF metabolites based on published data from mice and humans (Melum et al., 2025; Zhang et al., 2024). We found that 16 of our golden hamster metabolites were present in the MRCLinkdb and that five were significantly depleted during torpor. Of these, adenosine, particularly stands out owing to the presence of its receptor on tanycytes (Fig. 5A,B), and previous literature linking it to thermoregulation and hypometabolism, including sleep and hibernation (Drew et al., 2017; Jinka et al., 2011; Swoap et al., 2007, 2012; Xiao et al., 2019; Zhang et al., 2009). Pharmacological evidence links adenosine receptor 1 stimulation to entry into torpor (Jinka et al., 2011), but this is the first time adenosine has been measured in the CSF during a spontaneous T–A cycle. Contrary to expectation of high adenosine at entry to torpor, the complete adenosine abundance profile suggests increasing adenosine during arousal, high adenosine at the beginning of the interbout euthermic period (IBE), which then diminishes during IBE and entry to torpor (Fig. S3). However, adenosine measured in the CSF may not relate to adenosine abundance in the proximity to the as yet unidentified hypothalamic nuclei that may be responsible for adenosine receptor 1-mediated torpor entry. Tanycytes do express adenosine receptor 1 (Fig. 5B; *Adora1*) but they also express *Adora2a* and *Adora2b* (Fig. 5B), which couple to Gs signalling not Gi signalling, so the impact of increased adenosine is difficult to interpret without further study. Furthermore, caution must be advised when interpreting a result from one animal, even when the result is repeatable over two T–A cycles. It is, however, interesting to speculate why high adenosine may be observed in the CSF during arousal and early in IBE. One possible explanation may be the high sleep pressure at arousal from torpor leads to high adenosine and this reduces in IBE period during sleep (Daan et al., 1991; Larkin and Heller, 1999; Strijkstra and Daan, 1998). Another possible explanation is that tanycytes use ATP as a gliotransmitter (Dali et al., 2023; Frayling et al., 2011; Lazutkaite et al., 2017), and express exonucleases to breakdown ATP to adenosine (Kim et al., 2025). Therefore, it is possible that the increase in adenosine in the CSF observed during arousal and early in IBE, results from increased tanycyte ATP gliotransmission, extracellular breakdown to adenosine (Fig. 5D; *Nt5e*) and diffusion into the CSF either via gap junctions or via the tanycyte expressed bidirectional transporters of adenosine (Fig. 5C; *Scl29a2* and *Slc29a1*), but at present this is purely speculation.

The depletion of phenylalanine and tyrosine during torpor may relate to their roles in energy supply and neurotransmitter metabolism (Fernstrom and Fernstrom, 2007); furthermore, adaptation in these pathways has been noted in the liver of hibernating bats (Pan et al., 2013). Finally, carnitine appears to deplete over time in torpor (Fig. 4B), and the tanycytes possess genes for the transport of carnitine (Fig. 4C,D), it is possible that uptake of carnitine by the tanycytes over torpor may provide an energy source for tanycytes, via carnitines role to supply long-chain fatty acids into mitochondria for energy (Bremer, 1983). However, questions arise over the source of carnitine in the CSF and caution must be applied in interpretation as the annotation of this metabolite is uncertain (MSI level C). The tanycyte gene–metabolite matching analysis is preliminary, and therefore no conclusions can be drawn; however, it does at least raise the possibility that T–A cycling CSF metabolites are sensed, transported and processed by the tanycytes. But more work is required to determine whether tanycytes or cycling metabolites play a role in T–A cycle regulation.

In conclusion, we have demonstrated, for the first time, that it is possible to sample from the third ventricle in a hibernating golden hamster over two T–A cycles. We have developed an analysis pipeline capable of identifying accumulating and depleting metabolites during T–A cycling, which can be used to assess consistency across T–A cycles within animals and across animals. This study lays the foundations for future work to identify the metabolites potentially regulating the T–A cycle.

## Supplementary Material

10.1242/jexbio.251702_sup1 Supplementary information

Table S1. Metabolites that show a significant cross-correlation with body temperature, listed with other relevant statistics – See separate excel file.
