# Supplementary material for: Continuous sampling of cerebrospinal fluid from the third ventricle of the hypothalamus to identify accumulating or depleting metabolites during hibernation
Source: J Exp Biol. 2026 Sep 9;229(17):jeb251702. doi: 10.1242/jeb.251702 (PMC13615680; doi:10.1242/jeb.251702)
Supplement: Supplementary file 1 [file jexbio-229-251702-s1.pdf]

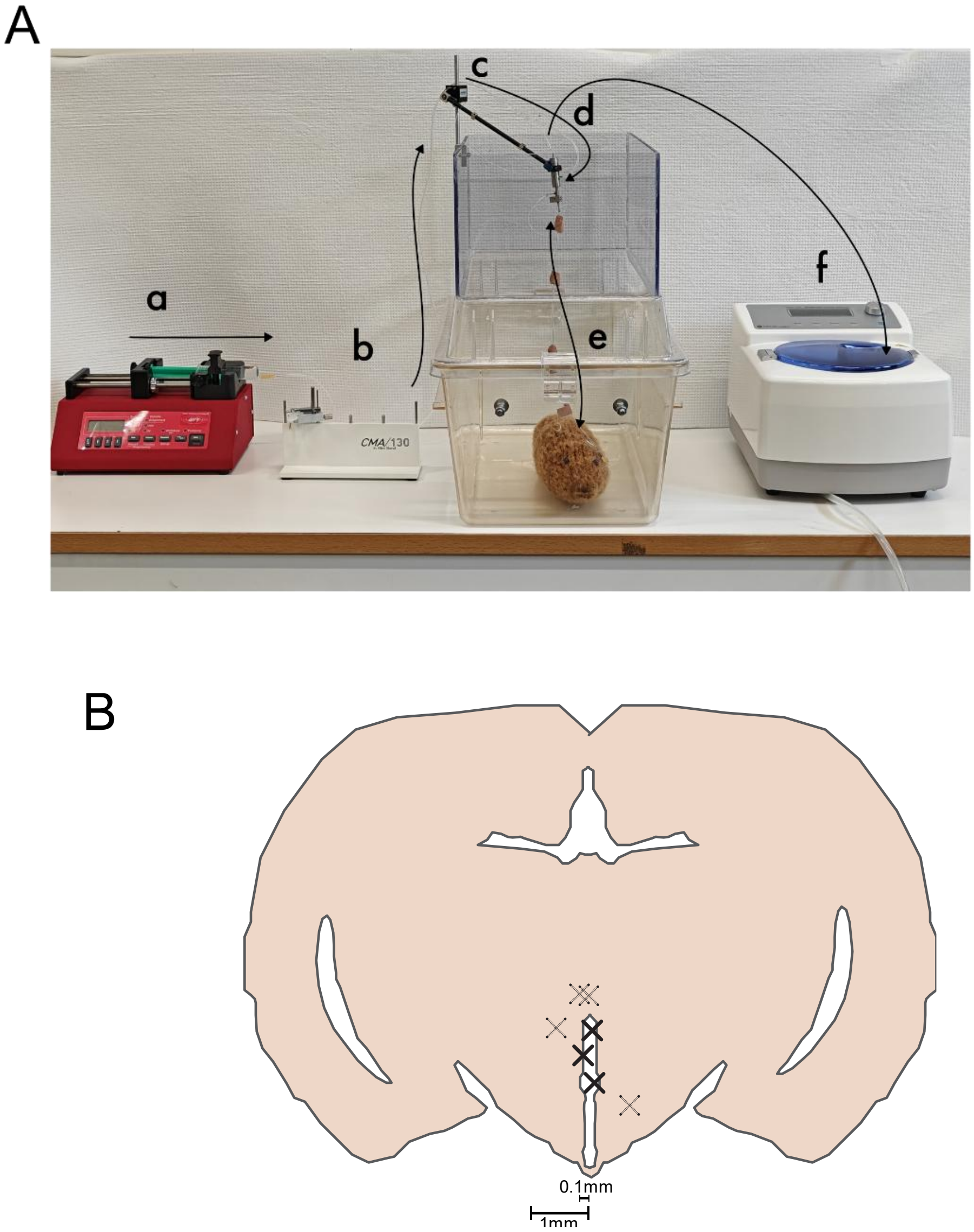

Fig. S1. Microdialysis home cage set-up

A. Components form left to right: a; aCSF dialysate perfusion pump set to 1 µL/min directs aCSF via b; FEP tubing to the c; swivel system mounted on top of the custom-built open lid. The swivel arm holds d; the multi axis counterbalance directing aCSF to e; the microdialysis probe cemented to the animal. Dialysate returns through the multi axis counterbalance to be collected in f; the refrigerated automated fraction collector. For parts and model details see methods section.

B. Placement of cannulas for all animals

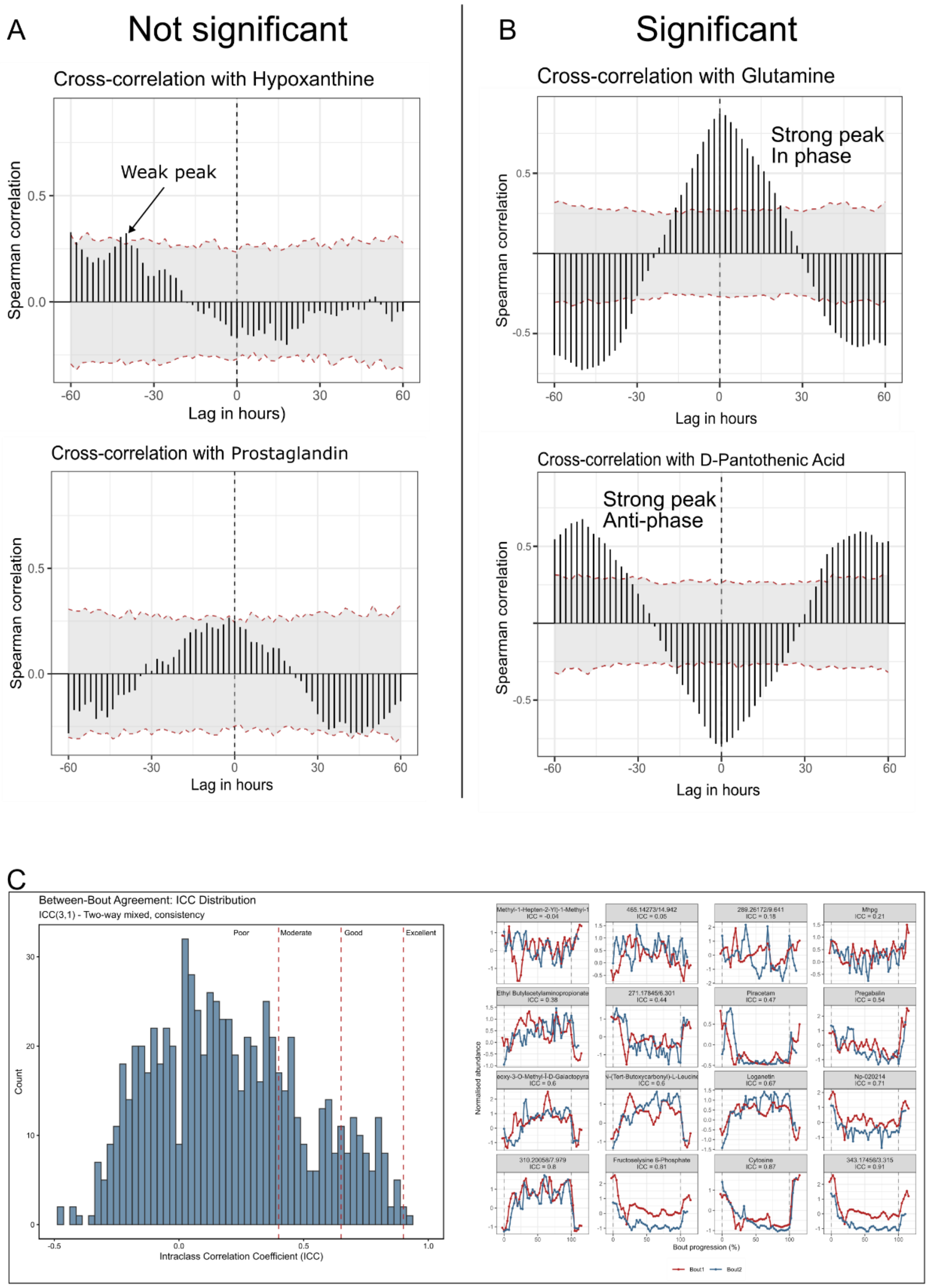
A
Not significant
Cross-correlation with Hypoxanthine
Weak peak
Spearman correlation
Lag in hours)
Cross-correlation with Prostaglandin
Spearman correlation
Lag in hours
B
Significant
Cross-correlation with Glutamine
Strong peak
In phase
Spearman correlation
Lag in hours
Cross-correlation with D-Pantothenic Acid
Strong peak
Anti-phase
Spearman correlation
Lag in hours
C
Between-Bout Agreement: ICC Distribution
ICC(3,1) - Two-way mixed, consistency
Poor
Moderate
Good
Excellent
Count
Intraclass Correlation Coefficient (ICC)
Normalised abundance
Bout progression (%)
Bout1
Bout2

Fig. S2. Cross-correlation significance testing example graphs

A. Shows two representative graphs of non-significant cross-correlations. Upper graph shows metabolite with weak peak formation around the permuted significance level. Lower graph shows unsignificant cross-correlating metabolite.

B. Shows two representative significant cross-correlations with clear peak formation around max correlation, indicating high correlation with body temperature. One in phase and one near antiphase example is given.

C. Left panel shows intra class coefficient score distribution for significant cross correlating metabolites. Right panels shows trace examples of ICC scores ranging from "Poor" (ICC < 0.4) to "Excellent" (ICC > 0.9)

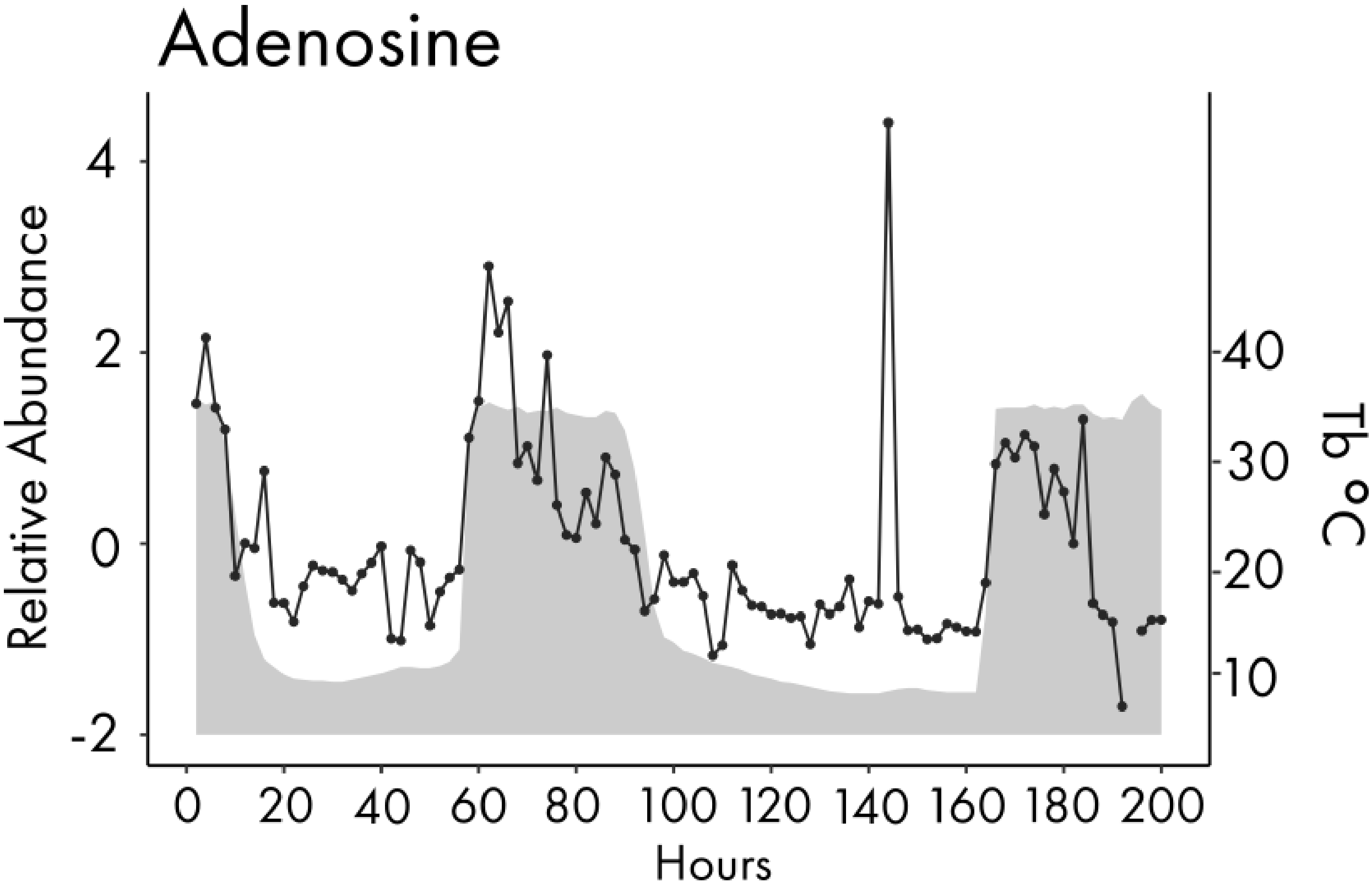

Fig. S3. Adenosine correlates with Tb but does not increase at torpor entry.

Trace of Adenosine (MSI A, ρ= 0.57, τ = -0.46, ICC = 0.83, p-adj = 0.02) shows that relative abundance correlates well with T_b (shaded area) where levels appear to increase at the start of arousal to diminish towards the next arousal.

Table S1. Metabolites that show a significant cross-correlation with body temperature, listed with other relevant statistics – See separate excel file.

Available for download at
https://journals.biologists.com/jeb/article-lookup/doi/10.1242/jeb.251702#supplementary-data
